# *In vitro* activity of aztreonam–avibactam and distribution of carbapenemase genes in ceftazidime–avibactam-resistant carbapenem-resistant Enterobacterales: data from the global Antimicrobial Testing Leadership and Surveillance, 2019–2023

**DOI:** 10.1128/aac.01549-25

**Published:** 2026-02-18

**Authors:** Yu-Lin Lee, Wei-Yao Wang, Yu-Tsung Huang, Mao-Wang Ho, Wen-Chien Ko, Po-Ren Hsueh

**Affiliations:** 1 Division of Infectious Disease, Department of Internal Medicine, Chung Shan Medical University Hospital 63276, Taichung, Taiwan; 2 School of Medicine, Chung Shan Medical University 34899, Taichung, Taiwan; 3 PhD Program in Medical Biotechnology, Institute of Genomics and Bioinformatics, National Chung-Hsing University 594230, Taichung, Taiwan; 4 Departments of Laboratory Medicine, National Taiwan University Hospital, National Taiwan University College of Medicine 38005, Taipei, Taiwan; 5 Department of Internal Medicine, National Taiwan University Hospital, National Taiwan University College of Medicine, Taipei, Taiwan; 6 Department of Internal Medicine, Division of Infectious Diseases, China Medical University Hospital, China Medical University 38020, Taichung, Taiwan; 7 Division of Infectious Diseases, Department of Internal Medicine, National Cheng Kung University Hospital 639603, Tainan, Taiwan; 8 Department of Medicine, College of Medicine, National Cheng Kung University 665116, Tainan, Taiwan; 9 Department of Laboratory Medicine, China Medical University Hospital, China Medical University 38020, Taichung, Taiwan; Entasis, Big Bay, Michigan, USA

**Keywords:** carbapenem-resistant Enterobacterales, ceftazidime–avibactam, aztreonam–avibactam, metallo-β-lactamase

## Abstract

Carbapenem-resistant Enterobacterales (CRE) pose significant treatment challenges. While ceftazidime–avibactam (CZA) is commonly used, resistance rates have been increasing. Aztreonam–avibactam (ATM–AVI) may represent a promising alternative. A total of 109,603 Enterobacterales isolates were collected from 307 sites across 63 countries between 2019 and 2023 as part of the Antimicrobial Testing Leadership and Surveillance (ATLAS) program. CRE were defined as isolates with a meropenem minimal inhibitory concentration (MIC) ≥2 mg/L. Susceptibility testing was conducted according to the Clinical and Laboratory Standards Institute (CLSI) 2025 guidelines, and β-lactamase genes were identified by multiplex PCR and sequencing. Of the total Enterobacterales isolates, 7,520 (7.6%) were identified as CRE, with *Klebsiella* species accounting for the majority (76.3%, 5,735/7,520). CZA susceptibility was 49.1% (3,696/7,520), with a significant increase in resistance from 42.1% (540/1,283) in 2019 to 61.0% (875/1,435) in 2023 (*P* < 0.05). Although CRE prevalence was highest in Asia (12.2%, 2,850/23,295), the highest rate of CZA resistance was observed in Africa/Middle East (73.9%, 557/754). In contrast, ATM–AVI demonstrated the highest *in vitro* activity, with 97.4% (7,324/7,520) of CRE isolates exhibiting MICs ≤4 mg/L. This activity remained strong against carbapenemase-producing strains, including metallo-β-lactamase (MBL) producers. However, reduced susceptibility was observed in *Escherichia* (80.2%, 556/693) and *Proteus* (88.9%, 56/63) species. Notably, resistance to ATM–AVI among carbapenem-resistant *Escherichia* species was geographically clustered in India, where 9.9% (36/364) of isolates were resistant. In conclusion, ATM–AVI exhibits potent activity against global CRE, including MBL producers, and outperforms other β-lactam/β-lactamase inhibitor combinations. However, emerging resistance in *Escherichia* and *Proteus* species—particularly with regional clustering—highlights the importance of continued global surveillance.

## INTRODUCTION

Carbapenem-resistant Enterobacterales (CRE) pose a major global health threat, with bloodstream infections associated with mortality rates of up to 50% and few effective treatment options (1). In 2019, antimicrobial resistance was estimated to account for 1.27 million deaths worldwide, with CRE recognized as one of the most critical pathogens (2, 3). Recent surveillance reports highlight alarming trends: in the European Union/European Economic Area, carbapenem-resistant *Klebsiella pneumoniae* bloodstream infections increased by 57.5% between 2019 and 2023, while in Latin America, CRE prevalence rose from 4.7% in 2018 to 12.9% in 2022 (3). Therapeutic options remain limited, as polymyxins and aminoglycosides are hindered by toxicity, whereas novel β-lactam/β-lactamase inhibitor (BLBLI) combinations—including ceftazidime–avibactam (CZA), imipenem–cilastatin–relebactam (I-R), and meropenem–vaborbactam (MVB)—are recommended by several international and regional guidelines for the treatment of severe CRE infections (4–6). Although those novel BLBLIs have expanded the therapeutic options against CRE, their effectiveness is constrained by the diversity of carbapenemase enzymes. Agents such as CZA, MVB, and I-R provide reliable coverage against class A carbapenemases (e.g., KPC) and, to some extent, class D enzymes (e.g., OXA-48-like). However, they lack activity against metallo-β-lactamase (MBL) producers, including NDM, VIM, and IMP, which are increasingly prevalent in Asia, the Middle East, and parts of South America (7, 8). In addition to newer BLBLIs, cefiderocol demonstrates potent *in vitro* activity against CRE, including isolates producing MBLs. However, susceptibility is consistently lower among NDM-producing strains than among those harboring VIM or IMP enzymes, with non-susceptibility in NDM lineages approaching 40% in some surveillance studies (9). Clinical data from CREDIBLE-CR and APEKS-NP show outcomes comparable to best available therapy, yet resistance emergence has been observed during treatment, particularly in NDM producers (10).

Aztreonam–avibactam (ATM–AVI) represents a promising therapeutic option against CRE. Aztreonam resists hydrolysis by MBLs but is inactivated by extended-spectrum β-lactamases (ESBLs), AmpC, and serine carbapenemases such as KPC and OXA-48-like. The addition of avibactam, a potent serine β-lactamase inhibitor, restores aztreonam’s activity (9). The REVISIT trial, which compared ATM–AVI to meropenem in the treatment of complicated intra-abdominal infections and hospital-acquired pneumonia, demonstrated superior clinical outcomes with a cure rate of 76.4% for ATM–AVI compared to 66.4% for meropenem, indicating its efficacy in these challenging infections (11). Furthermore, the ASSEMBLE trial, which evaluated ATM–AVI in treating serious infections caused by MBL-producing gram-negative bacteria, showed a clinical cure rate of 42% in the ATM–AVI group, while the best available therapy group had a cure rate of only 0%, highlighting the significant advantage of ATM–AVI in these difficult-to-treat (DTR) infections (12). In terms of regulatory approval, ATM–AVI was granted European Medicines Agency (EMA) approval in April 2024 and received approval from the U.S. Food and Drug Administration in February 2025 (13, 14). This rapid approval reflects the pressing need for new therapeutic options to combat the growing resistance of gram-negative bacteria to existing antibiotics.

While early clinical and regional surveillance studies show encouraging *in vitro* activity, current data remain fragmented and geographically restricted. Resistance mechanisms vary globally, with KPC prevalent in North America, OXA-48-like in Europe, and NDM in Asia, leaving the worldwide activity profile of ATM–AVI incompletely defined. This study analyzed data from the global Antimicrobial Testing Leadership and Surveillance (ATLAS) program (2019–2023) to evaluate the *in vitro* activity of ATM–AVI against CRE. By providing comprehensive susceptibility data across regions and carbapenemase genotypes, we aim to clarify the global role of ATM–AVI in addressing current therapeutic gaps and to inform its potential use in clinical practice.

## MATERIALS AND METHODS

### Bacterial collection of CRE isolates

Between 2019 and 2023, a total of 307 participating sites from 62 countries across 6 global regions—Africa/Middle East (14 countries), Asia (9), Europe (26), Latin America (10), North America (1), and Oceania (2)—contributed at least 1 Enterobacterales isolate. All clinical isolates were non-duplicate and comprised a pre-specified set of species collected from patients with documented clinical infections at each site. CRE were defined as Enterobacterales isolates exhibiting meropenem MICs of ≥2 mg/L in this study. The Enterobacterales genera included in this study were *Klebsiella* species, *Escherichia* species, *Enterobacter* species, *Proteus* species, *Serratia* species, *Citrobacter* species, *Morganella* species, *Providencia* species, *Raoultella* species, *Pantoea* species, *Pluralibacter* species, *Cronobacter* species, and *Lelliottia* species. The *Salmonella* species was excluded because the antimicrobial agents and susceptibility criteria recommended by the Clinical and Laboratory Standards Institute (CLSI) differ from those applied to other Enterobacterales (15). The number of species within each genus of Enterobacterales is summarized in Table S1. All isolates were shipped to the central reference laboratory (International Health Management Associates, Inc. [IHMA], Schaumburg, IL, USA) for identification using matrix-assisted laser desorption ionization–time of flight mass spectrometry (Bruker Biotyper, Bruker Daltonics, Billerica, MA, USA). Patient-level data, including outpatient vs inpatient status, ward or intensive care unit (ICU) location, culture source, and age, were collected. The annual distribution of isolates and participating sites by country is provided in Table S2. From October 9, 2025, onward, AMR surveillance data for sites in the United States are no longer accessible. Therefore, only Canada remains as the included country and site within North America. The ATLAS program received approval from the institutional review boards of the participating sites. National Taiwan University Hospital (Taipei, Taiwan) (NTUH 201211047RSC) served as a representative hospital in Taiwan. Given that the program focused on the collection of bacterial isolates and basic characteristic data with minimal risk for patients, the requirement for informed consent was waived.

### Antimicrobial susceptibility testing

Antimicrobial susceptibility was assessed for amikacin, aztreonam, ATM–AVI, cefepime, ceftazidime, CZA, ceftolozane–tazobactam (C/T), ciprofloxacin, colistin, gentamicin, levofloxacin, meropenem, MVB, piperacillin–tazobactam, tigecycline, and trimethoprim–sulfamethoxazole. Testing was performed with frozen broth microdilution panels prepared by the IHMA, following the procedures outlined by the CLSI. MICs were interpreted according to the CLSI 2025 criteria, applying parenteral breakpoints as reference standards (15). Because CLSI M100-Ed35 does not provide interpretive criteria for ATM–AVI against Enterobacterales, we adopted the European Committee on Antimicrobial Susceptibility Testing (EUCAST) recommendation and used an MIC ≤ 4 mg/L as the susceptibility threshold (16). Difficult-to-treat (DTR) phenotypes were characterized as isolates that were non-susceptible (intermediate or resistant according to CLSI) to all β-lactams, fluoroquinolones, and carbapenems, excluding CZA. The multidrug-resistant (MDR) phenotype of Enterobacterales was defined as isolates resistant to ≥1 compound (according to CLSI breakpoints) from at least three of the following antibiotic groups: aminoglycosides, carbapenems, cephalosporins, monobactams, quinolones, glycylcyclines (tigecycline by using FDA breakpoints), penicillins, tetracyclines, polymyxins, and trimethoprim-sulfamethoxazole.

### Detection of β-lactamase genes

Detection of β-lactamase genes was performed using the standardized and fully validated multiplex PCR assay panels developed by the IHMA. These assays target major β-lactamase families, including *bla*KPC, *bla*_NDM_, *bla*_VIM_, *bla*_IMP_, and *bla*_OXA-48-like_ carbapenemases; ESBL families (*bla*_SHV_, *bla*_TEM_, *bla*_CTX-M_, *bla*_VEB_, *bla*_PER_, and *bla*_GES_); and plasmid-mediated AmpC enzymes (ACC, ACT, CMY, DHA, FOX, MIR, and MOX). Primer sets were first verified *in silico* against reference sequences in the NCBI database. Assay validation was conducted at IHMA using a panel of well-characterized control strains representing each β-lactamase family, followed by assessment of analytical performance characteristics, including sensitivity, specificity, reproducibility, and inter-run consistency. All positive amplicons were further confirmed by Sanger sequencing, and the resulting sequences were aligned to NCBI reference gene sequences for definitive identification. These multiplex PCR methods and their validation have been described in detail in previous ATLAS publications (17, 18). Variants of carbapenemase genes were characterized by sequencing entire β-lactamase genes and aligning the results with reference sequences available in the NCBI database (www.ncbi.nlm.nih.gov). Other mechanisms that may contribute to carbapenem resistance—such as penicillin-binding protein (PBP) alterations, porin loss, and efflux pump upregulation—were not assessed in this study.

### Statistical analysis

All statistical evaluations were performed with MedCalc software, version 22.016 (MedCalc Software Ltd., Los Angeles, CA, USA). Comparisons of categorical data were carried out using either the chi-square test or Fisher’s exact test, as appropriate. A two-tailed approach was applied to all analyses, and results were considered statistically significant when *P* values were less than 0.05.

## RESULTS

### Characteristics of Enterobacterales isolates

Between 2019 and 2023, a total of 98,933 Enterobacterales isolates were collected from 63 countries worldwide, of which 7,520 (7.6%) were resistant to meropenem (MIC ≥ 2 mg/L) (Table S3). The prevalence of CRE among all Enterobacterales isolates was 5.3% in Europe, 12.2% in Asia, 10.5% in Latin America, 0.3% in North America, 7.3% in Africa/Middle East, and 0.1% in Oceania. The annual numbers of CRE remained relatively stable throughout the study period. Among the CRE, *Klebsiella* species accounted for the majority of isolates (*n* = 5,735, 76.3%), followed by *Escherichia* (*n* = 693, 9.2%) and *Enterobacter* (*n* = 495, 6.6%). Patient demographics and isolate characteristics are summarized in Table S3. Most CRE isolates (*n* = 6,699, 89.1%) were obtained from hospitalized adults, with nearly half of these patients admitted to intensive care units (ICUs; *n* = 3,102, 41.3%). Half of the study population was aged >60 years (*n* = 3,751, 49.9%), whereas pediatric patients represented only 6.4% (*n* = 485). The most common infection sources were respiratory tract infections (*n* = 2,061, 27.4%), bloodstream infections (*n* = 1,977, 26.3%), and genitourinary infections (*n* = 1,511, 20.1%). Additionally, 4,881 (63.7%) and 699 (9.3%) of the CRE isolates displayed an ESBL and AmpC β-lactamase genotypes. Nearly all CRE exhibited MDR (99.9%, 7,512/7,520) and DTR (94.5%, 7,109/7,520) phenotypes.

### ATM–AVI susceptibility and MIC distribution in CRE by genus, species, CZA resistance, and carbapenemase genes

The susceptibility of Enterobacterales isolates from different genera to various antimicrobial agents is summarized in Table 1. Genera *Morganella* (*n* = 15) and *Raoultella* (*n* = 1) were excluded from the analysis due to insufficient sample size (<30 isolates), which was deemed unrepresentative. Overall, the CRE isolates exhibited an MDR profile. Among the tested agents, only tigecycline (92.8%, 6,978/7,520) and ATM–AVI (97.4%, 7,325/7,520) demonstrated high susceptibility rates. In contrast, the activities of other novel BLBLIs were markedly lower, with susceptibility rates of 49.2% for CZA, 1.1% for C/T, and 32.5% for MVB. Non-susceptibility to third-generation and fourth-generation cephalosporins, fluoroquinolones, aztreonam, and piperacillin-tazobactam exceeded 90%. Among aminoglycosides, susceptibility rates were 33.5% for amikacin and 31.0% for gentamicin.

**TABLE 1:** Antimicrobial susceptibility of different genera of CRE isolates collected from the ATLAS program, 2019–2023^*c*^

CRE (no. of isolates)	% of isolates susceptible to:															
	AMK	ATM	ATM–AVI^*a*^	FEP	CAZ	CZA	C/T	CIP	COL^*b*^	GEN	LVX	IPM	MVB	TZP	TGC	TMP–SMX
Enterobacterales (7,520)	33.5	9.4	97.4	1.3	2.4	49.2	1.1	5.0	0.0	31.0	7.4	0.96	32.5	0.5	92.8	17.6
*Klebsiella* spp. (5,735)	29.7	5.3	99.5	1.0	1.9	57.9	1.0	3.6	0.0	29.1	5.2	1.0	35.3	0.2	93.8	17.4
*Escherichia* spp. (693)	46.6	8.5	80.2	0.6	0.6	14.0	1.0	5.5	0.0	45.7	7.2	0.9	12.7	0.9	99.0	11.1
*Enterobacter* spp. (495)	58.0	21.0	98.6	1.6	1.6	22.6	0.7	12.5	0.0	34.1	21.6	0.4	29.8	1.4	90.7	23.0
*Providencia* spp. (217)	17.5	62.2	94.5	3.7	1.4	8.3	1.1	3.2	0.0	15.2	3.2	0.5	15.4	1.8	75.6	11.1
*Citrobacter* spp. (126)	63.5	27.8	98.4	2.4	3.2	21.4	1.1	14.3	0.0	33.3	19.8	1.59	28.7	0.8	94.4	16.7
*Serratia* spp. (175)	38.3	16.6	98.3	7.4	24.6	61.1	4.7	20.0	0.0	49.7	34.3	0.6	53.0	2.3	77.7	46.3
*Proteus* spp. (63)	23.8	60.3	88.9	6.4	9.5	15.9	5.6	12.7	0.0	14.3	11.1	3.2	33.3	7.9	58.7	9.5

^*a*^ Isolates with ATM–AVI MIC ≤ 4 mg/L.

^*b*^ Isolates with colistin MIC ≤ 2 mg/L.

^*c*^ AMK, amikacin; ATM, aztreonam; ATM-AVI, aztreonam-avibactam; CAZ, ceftazidime; CIP, ciprofloxacin; COL, colistin; C/T, ceftolozane-tazobactam; CZA, ceftazidime-avibactam; FEP, cefepime; GEN, gentamicin; IPM, imipenem-cilastatin; LVX, levofloxacin; MVB, meropenem–vaborbactam; TGC, tigecycline; TMP-SMX, trimethoprim-sulfamethoxazole; TZP, piperacillin-tazobactam.

Table 2 illustrates the variability in CZA resistance among Enterobacterales, stratified by genus, study year, and geographic region. With the exception of *Raoultella*, which was represented by a single isolate, the highest rate of CZA resistance was observed in *Providencia* species (91.7%, 199/217), followed by *Escherichia* (86.0%, 596/693), *Proteus* (84.1%, 53/63), *Citrobacter* (78.6%, 99/126), *Enterobacter* (77.4%, 383/495), *Morganella* (60.0%, 9/15), *Klebsiella* (42.1%, 2,416/5,735), and *Serratia* (38.9%, 68/175) (*P* < 0.05). A temporal trend of increasing CZA resistance was evident, with resistance rates rising from 42.1% (540/1,283) in 2019 to 47.5% (705/1,485) in 2020, 48.9% (878/1,797) in 2021, 54.3% (826/1,520) in 2022, and reaching 61.0% (875/1,435) in 2023 (*P* < 0.05). Geographic analysis revealed the highest CZA resistance among CRE isolates from Africa/Middle East (73.9%, 557/754), followed by Asia (62.4%, 1,779/2,850), Latin America (43.4%, 673/1,551), North America (40.0%, 12/30), and Europe (34.3%, 801/2,332) (*P* < 0.05)

**TABLE 2:** The distribution of CRE with different susceptibility to CZA in the ATLAS study, 2019–2023^*b*^

Demographic parameter	No. (%) of CZA-resistant isolates (*n* = 3,824)	No. (%) of CZA-susceptible isolates (*n* = 3,696)
Genus^*a*^		
*Klebsiella*	2,416 (63.2)	3,319 (89.8)
*Escherichia*	596 (15.6)	97 (2.6)
*Enterobacter*	383 (10.0)	112 (3.0)
*Providencia*	199 (5.2)	18 (0.5)
*Citrobacter*	99 (2.6)	27 (0.7)
*Serratia*	68 (1.8)	107 (2.9)
*Proteus*	53 (1.4)	10 (0.3)
*Morganella*	9 (0.2)	6 (0.2)
*Raoultella*	1 (<0.1)	0 (0)
Year^*a*^		
2019	540 (14.1)	743 (20.1)
2020	705 (18.4)	780 (21.1)
2021	878 (23.0)	919 (24.9)
2022	826 (21.6)	694 (18.8)
2023	875 (22.9)	560 (15.2)
Continent^*a*^		
Asia	1,779 (46.5)	1,071 (29.0)
Europe	801 (20.9)	1,531 (41.4)
Latin America	673 (17.6)	878 (23.8)
Africa/Middle East	557 (14.6)	197 (5.3)
North America	12 (0.3)	18 (0.5)
Oceania	2 (0.1)	1 (<0.1)
Genotype		
ESBL^*a*^	2,649 (69.3)	2,163 (58.5)
AmpC	637 (16.7)	62 (1.7)
MBL positive^*a*^	3,433 (89.8)	27 (0.7)
IMP	23 (0.6)	3 (0.1)
NDM	2,186 (57.2)	18 (0.5)
VIM	116 (3.0)	5 (0.1)
IMP + NDM	7 (0.2)	0 (0)
NDM + VIM	7 (0.2)	0 (0)
IMP + KPC	2 (0.1)	0 (0)
NDM + KPC	80 (2.1)	1 (<0.1)
NDM + OXA-48	950 (24.8)	0 (0)
NDM + KPC + OXA-48	1 (<0.1)	0 (0)
VIM + KPC	53 (1.4)	0 (0)
VIM + OXA-48	2 (0.1)	0 (0)
NDM + VIM + KPC	6 (0.2)	0 (0)
CBPM-positive, MBL-negative^*a*^	52 (1.4)	2,958 (80)
KPC	26 (0.7)	1,730 (46.8)
OXA-48	25 (0.7)	1,224 (33.1)
KPC + OXA-48	1 (<0.1)	4 (0.1)

^*a*^ A *P* value calculated by chi-square test less than 0.05.

^*b*^ CBPM, carbapenemases; CZA, ceftazidime–avibactam; ESBL, extended-spectrum β-lactamase; MBL, metallo-β-lactamase.

ATM–AVI MIC distributions for common Enterobacterales species are presented in Table 3. Most species showed susceptibility rates at or above 95%; however, *Escherichia coli* (80.3%, 555/691) and *Proteus mirabilis* (88.3%, 53/60) exhibited slightly reduced activity. Among CZA-resistant CRE isolates, *E. coli*, *P. mirabilis*, and *Providencia rettgeri* demonstrated lower susceptibility rates (78.1% [464/594], 86.0% [43/50], and 89.5% [85/95], respectively) than other species of Enterobacterales. Notably, carbapenem-resistant *E. coli* isolates harboring detectable carbapenemase genes showed similar susceptibility to ATM–AVI compared to those lacking such genes (79.8% [470/589] vs 86.5% [85/102], *P* = 0.26). However, *P. mirabilis* with isolates carrying carbapenemase genes exhibited greater susceptibility than those without (93.5% [43/46] vs 71.4% [10/14], *P* = 0.024), although this finding should be interpreted cautiously due to the small sample size.

**TABLE 3:** MIC distributions of ATM–AVI against common species of Enterobacterales stratified by carbapenem resistance, CZA resistance, CBPM, and MBL^*b*^

Species (no. of isolates)	Cumulative percentage of isolates inhibited at ATM–AVI MIC (mg/L) of:													
	0.015	0.03	0.06	0.12	0.25	0.5	1	2	4^*c*^	8	16	32	64	128
CRE^*a*^														
*Citrobacter freundii* (95)	2.1	12.6	41.1	65.3	79.0	93.7	95.8	96.8	**97.9**	97.9	97.9	97.9	99.0	100
*Enterobacter cloacae* (190)	0.5	3.7	26.8	52.1	73.2	90.0	95.3	98.4	**99.5**	99.5	100			
*Enterobacter hormaechei* (164)		1.2	15.2	42.1	65.9	79.9	92.1	96.3	**98.2**	98.8	100			
*Enterobacter* spp. (107)	1.9	8.4	23.4	45.8	62.6	73.8	86.0	90.7	**97.2**	98.1	98.1	98.1	100	
*Escherichia coli* (691)	2.2	6.7	15.2	22.9	29.4	37.9	49.5	63.7	**80.3**	92.6	96.1	97.3	98.3	100
*Klebsiella oxytoca* (59)	3.4	11.9	22.0	52.5	72.9	94.9	94.9	96.6	**100**					
*Klebsiella pneumoniae* (5,568)	0.6	2.6	8.2	28.6	69.5	92.2	97.7	99.1	**99.6**	99.6	99.7	99.8	99.9	100
*Proteus mirabilis* (60)	51.7	55.0	58.3	66.7	73.3	75.0	78.3	78.3	**88.3**	90.0	95.0	95.0	95.0	100
*Providencia rettgeri* (105)	26.7	34.3	39.1	45.7	57.1	63.8	73.3	75.2	**90.5**	94.3	96.2	97.1	97.1	100
*Providencia stuartii* (96)	29.2	43.8	68.8	86.5	92.7	97.9	97.9	97.9	**99.0**	99.0	99.0	100		
*Serratia marcescens* (151)			9.9	47.7	70.2	86.8	93.4	97.4	**98.7**	98.7	98.7	100		
CZA-R in CRE														
*Citrobacter freundii* (79)	2.5	15.2	44.3	67.1	81.0	94.9	97.5	97.5	**97.5**	97.5	97.5	97.5	98.7	100
*Enterobacter cloacae* (139)	0.7	5.0	30.9	54.7	74.8	92.8	97.8	100						
*Enterobacter hormaechei* (144)		1.4	16.0	43.8	68.1	82.6	93.8	96.5	**97.9**	98.6	100			
*Enterobacter* spp. (81)	2.5	9.9	25.9	49.4	67.9	80.3	91.4	95.1	**96.3**	97.5	97.5	97.5	100	
*Escherichia coli* (594)	0.7	5.1	12.8	19.7	23.2	31.8	44.3	59.8	**78.1**	91.6	95.5	96.8	98.0	100
*Klebsiella oxytoca* (35)	2.9	11.4	25.7	60.0	80.0	100								
*Klebsiella pneumoniae* (2,344)	0.6	3.8	12.1	38.0	77.7	94.5	97.7	98.5	**99.1**	99.2	99.3	99.5	99.7	100
*Proteus mirabilis* (50)	52.0	56.0	60.0	68.0	74.0	76.0	78.0	78.0	**86.0**	88.0	94.0	94.0	94.0	100
*Providencia rettgeri* (95)	25.3	30.5	35.8	43.2	54.7	61.1	70.5	72.6	**89.5**	93.7	95.8	96.8	96.8	100
*Providencia stuartii* (88)	31.8	47.7	72.7	90.9	95.5	100								
*Serratia marcescens* (62)			11.3	48.4	61.3	80.7	90.3	96.8	**96.8**	96.8	96.8	100		
CBPM (+), MBL (−) in CRE														
*Citrobacter freundii* (12)			25.0	50.0	66.67	91.67	91.67	100						
*Enterobacter cloacae* (42)			14.3	50.0	76.2	90.5	97.6	100						
*Enterobacter hormaechei* (13)			7.7	38.5	53.9	69.2	92.3	100						
*Enterobacter* spp. (13)		7.7	23.1	61.5	69.2	69.2	100							
*Escherichia coli* (54)	1.9	9.3	29.6	48.2	72.2	75.9	77.8	81.5	**88.9**	100				
*Klebsiella oxytoca* (21)	4.8	9.5	14.3	38.1	61.9	85.7	85.7	90.5	**100**					
*Klebsiella pneumoniae* (2,696)	0.5	1.7	5.8	24.3	69.5	94.3	98.7	99.5	**99.8**	99.9	100			
*Proteus mirabilis* (6)	33.3	33.3	33.3	50.0	66.7	66.7	100							
*Providencia rettgeri* (8)	25.0	37.5	37.5	37.5	37.5	50.0	62.5	75.0	**87.5**	87.5	87.5	87.5	87.5	100
*Providencia stuartii* (4)			50.0	75.0	100									
*Serratia marcescens* (69)			10.1	47.8	76.8	91.3	95.7	98.6	**100**					
MBL (+) in CRE														
*Citrobacter freundii* (57)	3.5	17.5	45.6	63.2	79.0	94.7	96.5	96.5	**98.3**	98.3	98.3	98.3	98.3	100
*Enterobacter cloacae* (121)	0.8	3.3	32.2	56.2	73.6	92.6	97.5	100						
*Enterobacter hormaechei* (122)		1.6	18.0	43.4	67.2	82.0	93.4	96.7	**97.5**	98.4	100			
*Enterobacter* spp. (64)	1.6	9.4	26.6	50.0	68.8	82.8	95.3	98.4	**98.4**	100				
*Escherichia coli* (535)	1.5	5.1	13.1	19.3	22.6	31.8	43.7	59.8	**78.9**	93.1	97.2	98.7	99.3	100
*Klebsiella oxytoca* (29)		10.3	24.1	65.5	82.8	100								
*Klebsiella pneumoniae* (2,176)	0.6	3.8	12.3	38.3	79.3	96.6	98.9	99.4	**99.8**	99.8	99.9	99.9	99.9	100
*Proteus mirabilis* (40)	62.5	67.5	72.5	82.5	87.5	90.0	90.0	90.0	**92.5**	95.0	100			
*Providencia rettgeri* (79)	21.5	27.9	34.2	40.5	51.9	59.5	70.9	72.2	**89.9**	94.9	97.5	97.5	97.5	100
*Providencia stuartii* (86)	30.2	46.5	72.1	90.7	95.4	100								
*Serratia marcescens* (56)			12.5	50.0	64.3	82.1	92.9	100						

^*a*^ Species with fewer than 50 carbapenem-resistant isolates are not included.

^*b*^ CBPM, carbapenemases; CRE, carbapenem-resistant Enterobacterales; CZA, ceftazidime–avibactam; MBL, metallo-β-lactamase.

^*c*^ Bold values indicate the susceptible breakpoint for aztreonam–avibactam used in this study.

**Fig 1:**
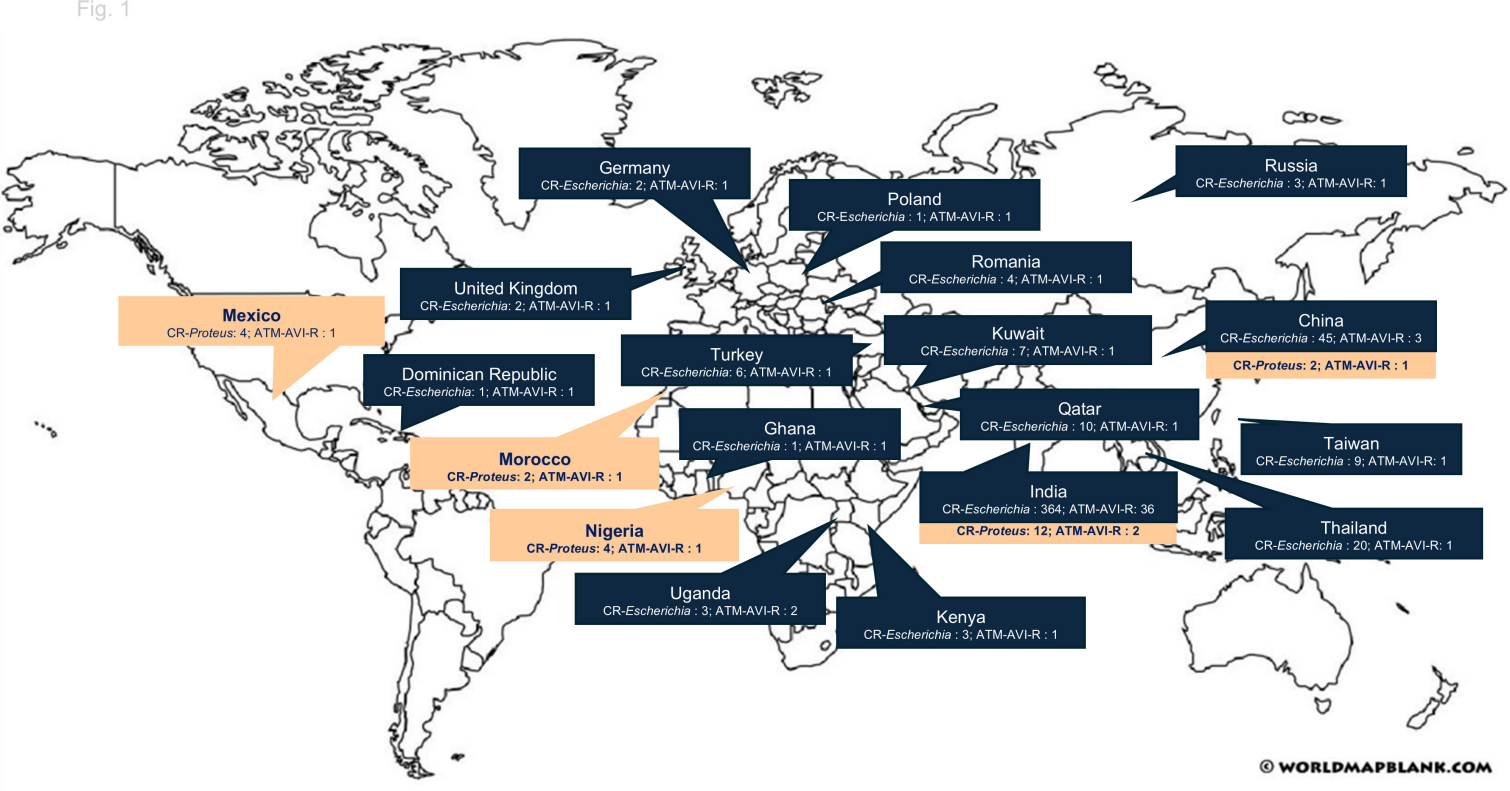
Geographic distribution of ATM–AVI-R carbapenem-resistant isolates belonging to *Escherichia coli* (CR-*Escherichia*) and *Proteus mirabilis* (CR-*Proteus*) collected worldwide in the ATLAS program, 2019–2023. Countries with CR-*Escherichia* and CR-*Proteus* but without ATM–AVI-R isolates are not shown, including Argentina, Australia, Belgium, Brazil, Canada, Colombia, Costa Rica, Croatia, Czech Republic, Denmark, France, Greece, Guatemala, Israel, Italy, Ivory Coast, Japan, Jordan, Latvia, Lithuania, Malawi, Malaysia, Panama, Philippines, South Africa, South Korea, Spain, Ukraine, United States, and Venezuela. Image source: World Map Blank (https://worldmapblank.com/).

The geographic distribution of 51 *E. coli* and 6 *P. mirabilis* isolates is shown in Fig. 1. India had the highest number of ATM–AVI-resistant (ATM–AVI-R) *Escherichia* species isolates, accounting for 70.6% (36/51). Among 364 CR *Escherichia* species isolates from India, 36 (9.9%) were resistant to ATM–AVI. In contrast, China contributed the second largest number of CR *Escherichia* species isolates (*n* = 45), and three (6.7%) exhibited resistance to ATM–AVI. This geographic difference may be attributed to variations in local resistance mechanisms, the spread of specific clonal populations, and differences in regional surveillance efforts. Interestingly, despite some countries reporting only sporadic CR *Escherichia* species isolates, a relatively high proportion of these isolates were resistant to ATM–AVI.

### β-Lactamase gene distribution in CRE by CZA resistance and region

In this study, among 7,520 meropenem-resistant Enterobacterales isolates, 6,470 (86.0%) carried at least 1 carbapenemase gene. The identified genes included *bla*_KPC_ (1,756), *bla*_OXA-48_ (1,249), *bla*_IMP_ (26), *bla*_NDM_ (2,204), *bla*_VIM_ (121), *bla*_KPC_ + *bla*_OXA-48_ (5), *bla*_IMP_ + *bla*_NDM_ (7), *bla*_NDM_ + *bla*_VIM_ (7), *bla*_IMP_ + *bla*_KPC_ (2), *bla*_NDM_ + *bla*_KPC_ (81), *bla*_NDM_ + *bla*_OXA-48_ (950), *bla*_VIM_ + *bla*_KPC_ (53), *bla*_VIM_ + *bla*_OXA-48_ (2), *bla*_NDM_ + *bla*_KPC_ + *bla*_OXA-48_ (1), and *bla*_NDM_ + *bla*_VIM_ + *bla*_KPC_ (6). The distribution of these carbapenemase genes according to CZA resistance is summarized in Table 2. Overall, 50.9% (3,824/7,520) of CRE isolates were resistant to CZA, of which 91.1% (3,485/3,824) were carbapenemase-producing (CP) strains. Analysis of carbapenemase genotypes revealed that CZA-resistant isolates were overwhelmingly dominated by MBL producers, consistent with the intrinsic inability of avibactam to inhibit class B enzymes. Among 3,824 CZA-resistant CRE, 89.8% (3,433/3,824) carried at least 1 MBL gene, whereas only 0.7% (27/3,696) of CZA-susceptible isolates harbored an MBL determinant. NDM was the most prevalent class B carbapenemase among CZA-resistant isolates, followed by VIM and IMP. In contrast, isolates harboring KPC or OXA-48-like enzymes constituted only 1.4% of the CZA-resistant group, underscoring the strong association between MBL production and phenotypic CZA resistance.

Among CZA-resistant isolates, ATM–AVI resistance remained rare, occurring in only 184 CRE isolates (2.4%). The detailed data are presented in Table S4. Unlike CZA resistance, which is almost entirely MBL-associated, ATM–AVI resistance showed a strong enrichment of AmpC enzymes, particularly CMY-type β-lactamases. Among ATM–AVI-resistant isolates that were also CZA resistant, 62.5% harbored AmpC enzymes, including CMY (32.6%) and DHA (8.7%), and 20.1% carried dual AmpC determinants (e.g., AMP + CMY). These findings suggest that overexpression or mutational enhancement of AmpC β-lactamases contributes to the degradation of aztreonam even in the presence of avibactam. Finally, the proportion of non-CP CRE was higher among CZA-susceptible isolates (19.2%, 711/3,696) compared with CZA-resistant isolates (8.9%, 339/3,824).

**Fig 2:**
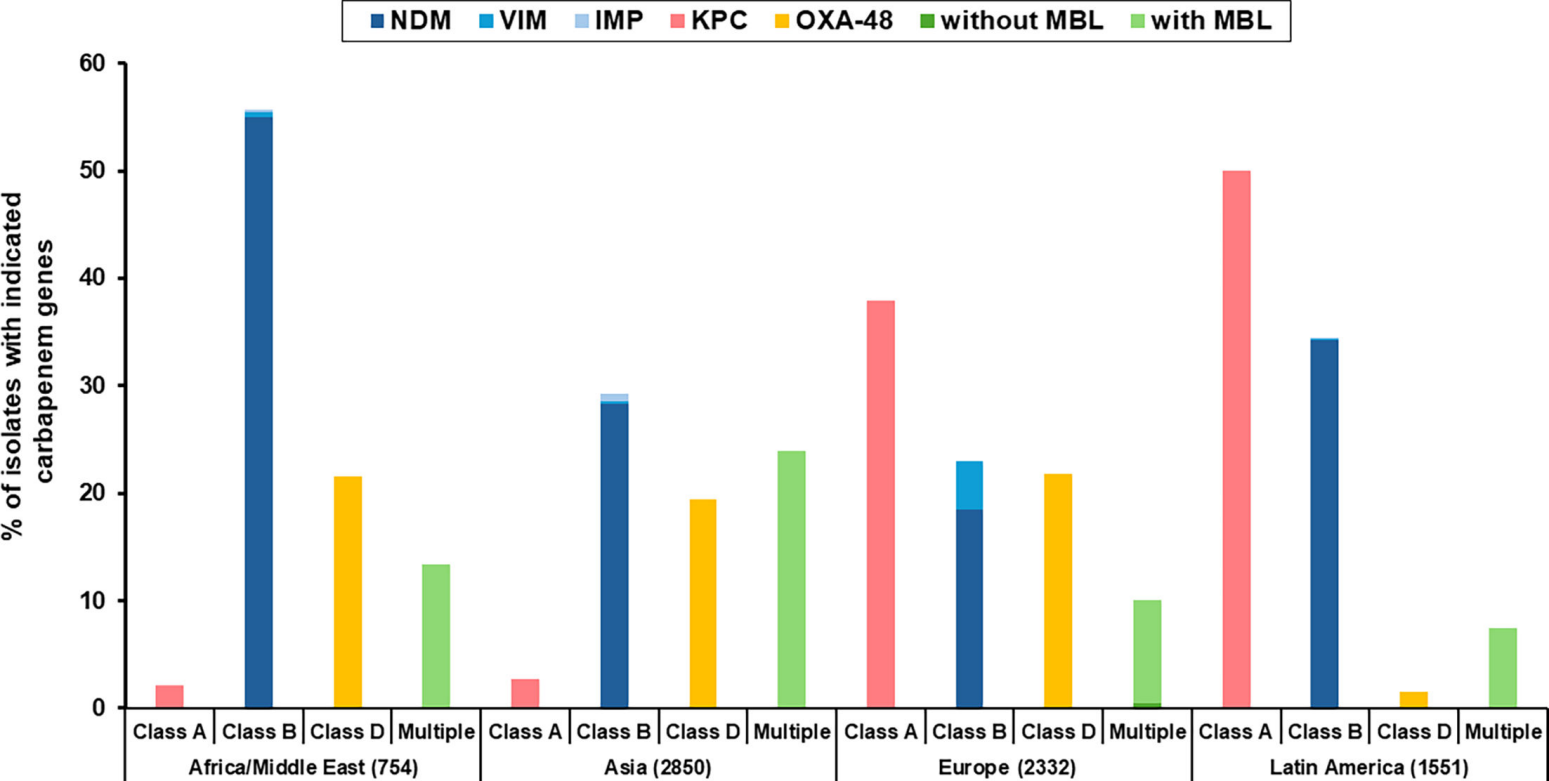
Global distribution of carbapenemase genes in CRE from the ATLAS program, 2019–2023. (Isolates from North America [*n* = 30] were omitted from this figure owing to the insufficient sample size and limited epidemiologic representativeness.)

Figure 2 illustrates the pronounced geographic variation in the distribution of carbapenemase genes among CRE worldwide. NDM-type carbapenemases were the predominant mechanism across Asia, with particularly high prevalence in South Asia and the Middle East. In contrast, OXA-48-like enzymes were most frequently detected in North Africa, the Middle East, and extended into parts of Southern and Eastern Europe. KPC-type carbapenemases predominated in the Americas, especially North America and South America, but were also commonly observed in Southern Europe, reflecting intercontinental spread. By comparison, VIM-type and IMP-type carbapenemases were less common globally and showed more localized distributions: VIM was mainly identified in Southern Europe and sporadically in Latin America, while IMP was observed in East Asia and selected regions of the Western Pacific. These findings reveal an east–west gradient in carbapenemase epidemiology, with NDM dominating in Asia, OXA-48-like in the Middle East and North Africa, and KPC in the Americas and Europe, whereas VIM and IMP remained regionally confined.

## DISCUSSION

This global surveillance study highlights the continuing threat of CRE, with isolates collected from 62 countries between 2019 and 2023. CRE prevalence remained stable overall but varied geographically, with the highest rates observed in Asia and Latin America. *Klebsiella* species predominated, followed by *Escherichia* and *Enterobacter*, reflecting their central role in global carbapenem resistance. Antimicrobial susceptibility testing confirmed that CRE isolates were extensively drug resistant, with >90% non-susceptibility to most standard agents. Among novel BLBLI combinations, CZA, C/T, and MVB demonstrated limited overall activity, consistent with the increasing prevalence of MBL producers. By contrast, ATM–AVI retained the highest potency, with >97% of CRE susceptible across regions. Although activity was reduced in the genera *Escherichia* and *Proteus*, ATM–AVI remained markedly superior to other BLBLIs, including in CZA-resistant isolates that harbor an MBL.

ATM–AVI has consistently demonstrated potent *in vitro* activity against CRE, particularly those producing MBLs. In a 5-year European study, 99.6%–99.7% of CRE isolates were susceptible (MIC ≤ 8 mg/L), with similar results in the United States, Latin America, Asia-Pacific, and Africa/Middle East regions (19). Another two global surveillance programs, SENTRY and ATLAS, also reported susceptibility rates exceeding 98%–99% among CRE isolates, with stable activity across Europe, Asia, and Latin America (17, 18). Importantly, ATM–AVI retained efficacy against MBL-producing and OXA-48-like-producing strains, where other recently approved BLBLIs such as CZA and MVB showed markedly reduced activity in regions with high MBL prevalence, particularly in the Asia-Pacific area (10, 20). Comparable findings have been confirmed in isolates from ICUs, where ATM–AVI remained the most active β-lactam agent against CRE (21). Our data are broadly consistent with these international observations, showing high activity of ATM–AVI against CRE, especially NDM-producing strains. Like surveillance findings, its efficacy was not compromised by isolate source (ICU vs non-ICU) or species distribution in most cases. Therefore, ATM–AVI has emerged as a promising therapeutic option for treating complicated infections caused by MDR gram-negative bacteria, particularly CRE and MBL-producing strains. In our study, ATM–AVI demonstrated similar activity against CZA-resistant isolates compared to CZA-susceptible isolates. This suggests that ATM–AVI could be considered a viable salvage therapy when first-line CZA treatment fails for CRE infections.

The diverse geographic distribution of carbapenemase genes presents a significant challenge in the treatment of CRE. The geographic variability in the distribution of carbapenemases, such as OXA-48-like enzymes in North Africa and Southern Europe, and NDM in South Asia, further complicates the use of CZA, MVB, and I-R, limiting its clinical application in areas with high MBL prevalence. In our study, we observed a high overall susceptibility of CRE isolates to ATM–AVI, with an impressive 98.7% of isolates showing susceptibility. These findings underscore ATM–AVI’s potential role as a therapeutic option in regions dominated by MBL-producing CRE, where other BLBLIs like CZA show limited efficacy against MBL producers. Given that ATM–AVI remains highly active even against MBL-producing strains, it could serve as a critical empiric or salvage therapy in regions with high MBL prevalence. Continuous regional surveillance and susceptibility testing remain essential to guide its optimal use, especially in settings with high carbapenemase gene variability.

Although CRE exhibit extremely low rates of resistance to ATM–AVI, we observed subtle differences in the MIC distribution, particularly among *E. coli* and *P. mirabilis*. Resistance to ATM–AVI in *E. coli* has emerged as a growing concern. Studies have shown that resistance mechanisms are multifactorial, primarily involving modifications in penicillin-binding protein 3 (PBP-3) and decreased outer membrane permeability. Notably, mutations in the *ftsI* gene, which encodes PBP-3, such as the insertion of four amino acids (YRIN/YRIK), have been directly linked to reduced susceptibility to ATM–AVI, especially in MBL producers (22, 23). These mutations result in structural changes to PBP-3, which is the primary target for aztreonam, thereby diminishing the drug’s effectiveness (24). The prevalence of resistance to ATM–AVI has been documented in several studies. Among a cohort of NDM-producing *E. coli* isolates, resistance to ATM–AVI was observed in approximately 40% of cases, with high-level resistance (MIC > 4 mg/L) seen in 19 isolates, primarily belonging to sequence types such as ST405, ST410, and ST361 (25, 26). These strains carried additional resistance determinants, including *bla*_CMY-42_, a type of AmpC β-lactamase, which further compounded the resistance. Moreover, while ATM–AVI retains efficacy against many MBL-producing strains, the emergence of PBP-3 mutations has led to treatment failure in some clinical settings, as seen in a liver transplant recipient who developed high-level resistance after treatment with CZA-ATM (24). Certain AmpC enzymes—most notably CMY and DHA—may further contribute to reduced ATM–AVI susceptibility, as their overexpression or structural modification can enable aztreonam hydrolysis even in the presence of avibactam (27, 28). These effects may act in combination with decreased outer-membrane permeability or increased efflux, which could account for the higher rates of ATM–AVI resistance seen in species such as *E. coli* and *Proteus* spp. Although direct mechanistic data for *Proteus mirabilis* remain limited, our findings similarly indicate that ATM–AVI resistance in this species likely involves pathways other than carbapenemase production.

Resistance of other genera of Enterobacterales is less common than in *Escherichia* species. In *K. pneumoniae*, resistance typically requires a combination of mechanisms including overexpression of AmpC-type β-lactamases (e.g., DHA-1), acquisition of ESBLs such as PER-2, and porin loss or truncation (OmpK35/OmpK36) (29). Besides, mutations in KPC-2 (e.g., Ser109Pro) and increased copy number of *bla*_KPC_ can further reduce susceptibility, especially when combined with porin deficiency and efflux pump overexpression (30). For the *Enterobacter cloacae* complex and *Klebsiella aerogenes*, resistance is mainly associated with hyperproduction of chromosomal AmpC β-lactamase, sometimes exceeding 500-fold over baseline, and porin alterations. Unlike *E. coli*, PBP3 insertions are not a major driver, and the specific amino acid changes in AmpC or PBP-3 are less well characterized (17, 31, 32).

Despite the comprehensive global scope of this surveillance, several limitations should be acknowledged. First, the analysis was restricted to *in vitro* susceptibility data and therefore does not account for pharmacokinetic/pharmacodynamic variability, host immune status, or treatment outcomes, which are essential to translate laboratory findings into clinical practice. Second, while ATM–AVI demonstrated consistently high activity overall, resistance was disproportionately observed in *E. coli* and *Proteus* species, yet the underlying molecular mechanisms were not systematically investigated in this dataset. Given that prior studies have linked PBP-3 insertions, ESBLs such as CTX-M-15, and AmpC overproduction (e.g., CMY-42) to ATM–AVI resistance, the absence of detailed genomic and functional validation represents an important gap. Furthermore, the geographic clustering of resistant *E. coli* isolates in South Asia, particularly India, underscores the need for region-specific molecular epidemiology and longitudinal monitoring to determine whether resistance is sporadic or indicative of expanding clonal lineages. Third, the lack of clinical correlation, including patient-level treatment and outcome data, highlights the necessity for prospective clinical trials and real-world effectiveness studies to better define the therapeutic role and stewardship considerations of ATM–AVI in the management of CRE infections. Finally, the ATLAS program includes only agents that are globally available and standardized across all participating laboratories. Several important antibiotics—such as ertapenem, cefiderocol, imipenem-relebactam, cefepime-zidebactam, and cefepime-taniborbactam—were not tested, which limits the breadth of comparative analyses.

In conclusion, this global surveillance study demonstrates that ATM–AVI retains potent *in vitro* activity against CRE, including those harboring MBL, and outperforms other recently approved BLBLI combinations. While overall susceptibility rates exceeded 98%, reduced activity was noted among *Escherichia* species and *Proteus* species, highlighting species-specific vulnerabilities that may signal early resistance trends. The geographic clustering of resistant isolates, particularly in India, further emphasizes the importance of continuous monitoring and molecular characterization to track emerging resistance mechanisms. These findings underscore the need for regionally tailored surveillance and clinical studies to optimize ATM–AVI use in diverse healthcare settings.

## SUPPLEMENTAL MATERIAL

Supplemental material aac.01549-25-s0001.docx Tables S1 to S4.

ASM does not own the copyrights to Supplemental Material that may be linked to, or accessed through, an article. The authors have granted ASM a non-exclusive, world-wide license to publish the Supplemental Material files. Please contact the corresponding author directly for reuse.
