# Supplementary material for: In vitro activity of aztreonam–avibactam and distribution of carbapenemase genes in ceftazidime–avibactam-resistant carbapenem-resistant Enterobacterales: data from the global Antimicrobial Testing Leadership and Surveillance, 2019–2023
Source: Antimicrob Agents Chemother. 2026 Feb 18;70(4):e01549-25. doi: 10.1128/aac.01549-25 (PMC13041386; doi:10.1128/aac.01549-25)
Supplement: Supplemental material — Tables S1 to S4. [file aac.01549-25-s0001.docx]

**TABLE S1** Number and percentage of species and isolates exhibiting carbapenem resistance among Enterobacterales genera in this study

| **Genus** | **Species** | **No. (%) of**  **all isolates** | **No. (%) of CR isolates** |
| --- | --- | --- | --- |
| *Citrobacter* | *Citrobacter amalonaticus* | 71 (0.1) | 2 (< 0.1) |
|  | *Citrobacter braakii* | 268 (0.3) | 2 (< 0.1) |
|  | *Citrobacter farmeri* | 33 (< 0.1) | 1 (< 0.1) |
|  | *Citrobacter freundii* | 1960 (2) | 95 (1.3) |
|  | *Citrobacter gillenii* | 8 (< 0.1) | 0 (< 0.1) |
|  | *Citrobacter koseri* | 2083 (2.1) | 12 (0.2) |
|  | *Citrobacter murliniae* | 2 (< 0.1) | 0 (< 0.1) |
|  | *Citrobacter sedlakii* | 46 (< 0.1) | 4 (0.1) |
|  | *Citrobacter* spp. | 289 (0.3) | 10 (0.1) |
|  | *Citrobacter youngae* | 2 (< 0.1) | 0 (< 0.1) |
| *Cronobacter* | *Cronobacter spp* | 1 (< 0.1) | 0 (< 0.1) |
| *Enterobacter* | *Enterobacter asburiae* | 188 (0.2) | 5 (0.1) |
|  | *Enterobacter bugandensis* | 791 (0.8) | 16 (0.2) |
|  | *Enterobacter cloacae* | 3968 (4) | 190 (2.5) |
|  | *Enterobacter hormaechei* | 2074 (2.1) | 164 (2.2) |
|  | *Enterobacter kobei* | 186 (0.2) | 2 (< 0.1) |
|  | *Enterobacter ludwigii* | 150 (0.2) | 0 (< 0.1) |
|  | *Enterobacter roggenkampii* | 131 (0.1) | 3 (< 0.1) |
|  | *Enterobacter* spp. | 2471 (2.5) | 107 (1.4) |
|  | *Enterobacter xiangfangensis* | 154 (0.2) | 8 (0.1) |
| *Escherichia* | *Escherichia coli* | 30115 (30.4) | 691 (9.2) |
|  | *Escherichia* spp. | 87 (0.1) | 2 (< 0.1) |
| *Klebsiella* | *Klebsiella aerogenes* | 2426 (2.5) | 40 (0.5) |
|  | *Klebsiella oxytoca* | 4071 (4.1) | 59 (0.8) |
|  | *Klebsiella pneumoniae* | 29676 (30) | 5568 (74) |
|  | *Klebsiella* spp. | 299 (0.3) | 42 (0.6) |
|  | *Klebsiella variicola* | 1665 (1.7) | 26 (0.3) |
| *Lelliottia* | *Lelliottia amnigena* | 1 (< 0.1) | 0 (< 0.1) |
| *Morganella* | *Morganella morganii* | 2996 (3) | 15 (0.2) |
|  | *Morganella* spp. | 5 (< 0.1) | 0 (< 0.1) |
| *Pantonea* | *Pantoea agglomerans* | 2 (< 0.1) | 0 (< 0.1) |
| *Pluralibacter* | *Pluralibacter gergoviae* | 2 (< 0.1) | 0 (< 0.1) |
| *Proteus* | *Proteus hauseri* | 130 (0.1) | 0 (< 0.1) |
|  | *Proteus mirabilis* | 3876 (3.9) | 60 (0.8) |
|  | *Proteus penneri* | 23 (< 0.1) | 0 (< 0.1) |
|  | *Proteus* spp. | 259 (0.3) | 1 (< 0.1) |
|  | *Proteus vulgaris* | 1185 (1.2) | 2 (< 0.1) |
| *Providenca* | *Providencia alcalifaciens* | 19 (< 0.1) | 0 (< 0.1) |
|  | *Providencia rettgeri* | 1001 (1) | 105 (1.4) |
|  | *Providencia rustigianii* | 3 (< 0.1) | 0 (< 0.1) |
|  | *Providencia* spp. | 121 (0.1) | 16 (0.2) |
|  | *Providencia stuartii* | 1016 (1) | 96 (1.3) |
| *Raoltella* | *Raoultella ornithinolytica* | 26 (< 0.1) | 1 (< 0.1) |
|  | *Raoultella planticola* | 4 (< 0.1) | 0 (< 0.1) |
|  | *Raoultella* spp. | 3 (< 0.1) | 0 (< 0.1) |
| *Serratia* | *Serratia liquefaciens* | 11 (< 0.1) | 0 (< 0.1) |
|  | *Serratia marcescens* | 4351 (4.4) | 151 (2) |
|  | *Serratia nematodiphila* | 1 (< 0.1) | 0 (< 0.1) |
|  | *Serratia odorifera* | 1 (< 0.1) | 0 (< 0.1) |
|  | *Serratia rubidaea* | 1 (< 0.1) | 0 (< 0.1) |
|  | *Serratia* spp. | 672 (0.7) | 24 (0.3) |
|  | *Serratia ureilytica* | 9 (< 0.1) | 0 (< 0.1) |

CR, carbapenem-resistant

**TABLE S2** Years and number of sites contributing Data of participating countries in the Antimicrobial Testing Leadership and Surveillance (ATLAS) program, 2019–2023.

| **Country** | **2019** | **2020** | **2021** | **2022** | **2023** |
| --- | --- | --- | --- | --- | --- |
| **Africa/Middle East** | | | | | |
| Cameroon | 0 | 1 | 1 | 1 | 1 |
| Ghana | 0 | 0 | 2 | 2 | 2 |
| Israel | 4 | 3 | 3 | 3 | 3 |
| Ivory Coast | 0 | 1 | 1 | 1 | 1 |
| Jordan | 0 | 1 | 1 | 1 | 1 |
| Kenya | 0 | 0 | 2 | 2 | 2 |
| Kuwait | 3 | 3 | 3 | 3 | 3 |
| Malawi | 0 | 0 | 1 | 2 | 2 |
| Morocco | 3 | 3 | 3 | 3 | 3 |
| Nigeria | 3 | 3 | 3 | 3 | 3 |
| Qatar | 1 | 1 | 1 | 1 | 1 |
| Saudi Arabia | 1 | 1 | 1 | 0 | 0 |
| South Africa | 4 | 4 | 4 | 2 | 3 |
| Uganda | 0 | 0 | 2 | 2 | 2 |
| **Asia** | | | | | |
| China | 17 | 4 | 12 | 13 | 13 |
| Hong Kong | 1 | 2 | 2 | 0 | 0 |
| India | 9 | 9 | 10 | 10 | 10 |
| Japan | 3 | 3 | 3 | 3 | 3 |
| Korea, South | 4 | 4 | 4 | 4 | 4 |
| Malaysia | 2 | 2 | 3 | 3 | 3 |
| Philippines | 3 | 3 | 3 | 3 | 3 |
| Taiwan | 4 | 4 | 4 | 4 | 4 |
| Thailand | 4 | 4 | 4 | 3 | 4 |
| **Europe** | | | | | |
| Belgium | 6 | 6 | 6 | 4 | 5 |
| Bulgaria | 0 | 0 | 0 | 1 | 1 |
| Croatia | 4 | 4 | 3 | 4 | 4 |
| Czech Republic | 4 | 4 | 4 | 4 | 4 |
| Denmark | 1 | 1 | 1 | 1 | 1 |
| Finland | 1 | 1 | 1 | 1 | 1 |
| France | 10 | 10 | 10 | 10 | 9 |
| Germany | 10 | 9 | 7 | 8 | 10 |
| Greece | 3 | 4 | 3 | 2 | 2 |
| Hungary | 3 | 4 | 2 | 3 | 4 |
| Ireland | 2 | 2 | 2 | 2 | 2 |
| Italy | 9 | 9 | 10 | 12 | 11 |
| Latvia | 1 | 1 | 1 | 1 | 0 |
| Lithuania | 2 | 2 | 2 | 2 | 2 |
| Netherlands | 2 | 2 | 1 | 1 | 1 |
| Poland | 4 | 4 | 3 | 3 | 4 |
| Portugal | 4 | 4 | 4 | 4 | 3 |
| Romania | 2 | 3 | 3 | 4 | 3 |
| Russia | 8 | 0 | 0 | 0 | 0 |
| Slovenia | 0 | 1 | 1 | 1 | 1 |
| Spain | 11 | 11 | 11 | 10 | 11 |
| Sweden | 1 | 1 | 1 | 1 | 1 |
| Switzerland | 2 | 2 | 2 | 2 | 2 |
| Turkey | 4 | 4 | 5 | 6 | 5 |
| Ukraine | 1 | 3 | 3 | 1 | 2 |
| United Kingdom | 6 | 4 | 4 | 4 | 5 |
| **Latin America** | | | | | |
| Argentina | 3 | 3 | 3 | 3 | 3 |
| Brazil | 6 | 8 | 7 | 6 | 5 |
| Chile | 3 | 3 | 3 | 3 | 3 |
| Colombia | 5 | 5 | 5 | 5 | 4 |
| Costa Rica | 1 | 1 | 1 | 1 | 1 |
| Dominican Republic | 1 | 1 | 1 | 1 | 1 |
| Guatemala | 2 | 2 | 2 | 2 | 2 |
| Mexico | 6 | 5 | 5 | 5 | 4 |
| Panama | 2 | 2 | 2 | 2 | 2 |
| Venezuela | 2 | 2 | 2 | 2 | 2 |
| **North America** | | | | | |
| Canada | 8 | 7 | 7 | 7 | 7 |
| **Oceania** | | | | | |
| Australia | 4 | 4 | 4 | 4 | 4 |
| New Zealand | 1 | 2 | 2 | 2 | 2 |

**TABLE S3** Patient demographics and culture source for Enterobacterales isolates collected in the ATLAS surveillance study, 2019 to 2023.

| **Demographic parameter** | **No. (%) of Enterobacterales**  **(n = 98,933)** | **No. (%) of CRE**  **(n = 7,520)** |
| --- | --- | --- |
| **Genus** |  |  |
| *Klebsiella* | 38137 (38.6) | 5735 (76.3) |
| *Escherichia* | 30202 (30.5) | 693 (9.2) |
| *Enterobacter* | 10113 (10.2) | 495 (6.6) |
| *Proteus* | 5473 (5.5) | 217 (2.9) |
| *Serratia* | 5046 (5.1) | 175 (2.3) |
| *Citrobacter* | 4762 (4.8) | 126 (1.7) |
| *Morganella* | 3001 (3) | 63 (0.8) |
| *Providencia* | 2160 (2.2) | 15 (0.2) |
| *Raoultella* | 33 (<0.1) | 1 (<0.1) |
| *Pantoea* | 2 (<0.1) | 0 (0) |
| *Pluralibacter* | 2 (<0.1) | 0 (0)- |
| *Cronobacter* | 1 (<0.1) | 0 (0)- |
| *Lelliottia* | 1 (<0.1) | 0 (0) |
| **Year** |  |  |
| 2019 | 19078 (19.3) | 1283 (17.1) |
| 2020 | 20296 (20.5) | 1485 (19.8) |
| 2021 | 21879 (22.1) | 1797 (23.9) |
| 2022 | 20599 (20.8) | 1520 (20.2) |
| 2023 | 17081 (17.3) | 1435 (19.1) |
| **Continent** |  |  |
| Europe | 44275 (44.8) | 2332 (31) |
| Asia | 23295 (23.6) | 2850 (37.9) |
| Latin America | 14790 (15) | 1551 (20.6) |
| North America | 10312 (10.4) | 30 (0.4) |
| Africa / Middle East | 3354 (3.4) | 754 (10) |
| Oceania | 2907 (2.9) | 3 (<0.1) |
| **Specimen source** |  |  |
| Circulatory | 25686 (26) | 1977 (26.3) |
| Respiratory | 22000 (22.2) | 2061 (27.4) |
| Genitourinary | 21740 (22) | 1511 (20.1) |
| Skin / musculoskeletal | 16771 (17) | 1133 (15.1) |
| Intestinal | 12545 (12.7) | 823 (10.9) |
| None given | 179 (0.2) | 14 (0.2) |
| Nervous system | 7 (<0.1) | 1 (<0.1) |
| Head, eye, ear, nose, throat | 4 (<0.1) | 0(0) |
| Instruments | 1 (<0.1) | 0(0) |
| **Ward** |  |  |
| Medicine general | 36767 (37.2) | 2489 (33.1) |
| Surgery general | 17016 (17.2) | 911 (12.1) |
| Emergency room | 12938 (13.1) | 490 (6.5) |
| Medicine ICU | 11868 (12) | 1751 (23.3) |
| Surgery ICU | 5607 (5.7) | 684 (9.1) |
| Pediatric general | 3518 (3.6) | 197 (2.6) |
| General unspecified ICU | 3241 (3.3) | 379 (5) |
| Others | 2966 (3) | 156 (2.1) |
| Pediatric ICU | 2763 (2.8) | 288 (3.8) |
| None given | 2249 (2.3) | 175 (2.3) |
| **Age** |  |  |
| 0 - 17 | 7666 (7.8) | 535 (7.1) |
| 18 - 30 | 5329 (5.4) | 453 (6) |
| 31 - 60 | 29582 (29.9) | 2627 (34.9) |
| 61+ | 54095 (54.7) | 3751 (49.9) |
| NA | 2261 (2.3) | 154 (2.1) |
| **Phenotype and genotype** |  |  |
| MDR (CLSI) | 40619 (41.1) | 7512 (99.9) |
| ESBL | 14174 (14.3) | 4812 (64) |
| AmpC | 1875 (1.9) | 699 (9.3) |
| DTR | 9219 (9.3) | 7109 (94.5) |
| CRE | 7520 (7.6) | 7520 (100) |
| MBL-Positive | 3671 (3.7) | 3460 (46) |
| CBPM Positive MBL-negative | 3600 (3.6) | 3010 (40) |

CBPM, carbapenemase; CRE, carbapenem-resistant Enterobacterales; ICU, intensive care units; MBL, metallo-beta-lactamase; MDR, multidrug-resistant; ESBL, extended-spectrum -lactamase; DTR, difficult-to-treat

**Table S4** The distribution of carbapenem-resistant Enterobacterales with different susceptibility ceftazidime-avibactam (CZA) and aztreonam-avibactam (ATM-AVI) in the ATLAS study, 2019-2023.

| **Demographic parameter** | **No. (%) of isolates with CZA-resistant and ATM-AVI-resistant**  **(n=184)** | **No. (%) of isolates with CZA-resistant and ATM-AVI-susceptible (n=3640)** |
| --- | --- | --- |
| **Genus** |  |  |
| *Klebsiella* | 24 (13) | 2392 (65.7) |
| *Escherichia* | 131 (71.2) | 465 (12.8) |
| *Enterobacter* | 6 (3.3) | 377 (10.4) |
| *Proteus* | 7 (3.8) | 188 (5.2) |
| *Serratia* | 3 (1.6) | 97 (2.7) |
| *Citrobacter* | 2 (1.1) | 0 (0) |
| *Morganella* | 0 (0) | 9 (0.3) |
| *Providencia* | 11 (6) | 188 (5.2) |
| *Raoultella* | 0 (0) | 1 (<0.1) |
| **Year** |  |  |
| 2019 | 12 (6.5) | 528 (14.5) |
| 2020 | 28 (15.2) | 677 (18.6) |
| 2021 | 36 (19.6) | 842 (23.1) |
| 2022 | 57 (31) | 769 (21.1) |
| 2023 | 51 (27.7) | 824 (22.6) |
| **Continent** |  |  |
| Europe | 11 (6) | 790 (21.7) |
| Asia | 153 (83.2) | 1626 (44.7) |
| Latin America | 4 (2.2) | 669 (18.4) |
| North America | 1 (0.5) | 11 (0.3) |
| Africa / Middle East | 15 (8.2) | 542 (14.9) |
| Oceania | 0 (0) | 2 (0.1) |
| **Phenotype and genotype** |  |  |
| ESBL | 54 (29.4) | 2595 (71.3) |
| AmpC | 115 (62.5) | 522 (14.3) |
| ACC | 0 (0) | 7 (0.2) |
| AMP | 0 (0) | 0 (0) |
| CMY | 60 (32.6) | 288 (7.9) |
| DHA | 16 (8.7) | 64 (1.8) |
| DOX | 0 (0) | 1 (<0.1) |
| AMP+CMY | 37 (20.1) | 123 (3.4) |
| AMP+DHA | 2 (1.1) | 28 (0.8) |
| CMY+DHA | 0 (0) | 7 (0.2) |
| ACC+CMY | 0 (0) | 1 (<0.1) |
| AMP+CMY+DHA | 0 (0) | 3 (<0.1) |
| MBL-Positive | 136 (73.9) | 3297 (90.6) |
| CBPM Positive MBL-negative | 5 (2.7) | 47 (1.3) |

CBPM, carbapenemase; MBL, metallo-beta-lactamase; ESBL, extended-spectrum -lactamase;
